# The social and personality neuroscience of empathy for pain and touch

**DOI:** 10.3389/fnhum.2013.00393

**Published:** 2013-07-26

**Authors:** Ilaria Bufalari, Silvio Ionta

**Affiliations:** ^1^ Dipartimento di Psicologia, Sapienza Università di RomaRoma, Italy; ^2^ Laboratorio di Neuroscienze Sociali, IRCCS Fondazione Santa LuciaRoma, Italy; ^3^ Rehabilitation Engineering Laboratory, Institute of Robotics and Intelligent Systems, Swiss Federal Institute of Technology (ETHZ)Zurich, Switzerland

**Keywords:** empathy, personality, social cognition, somatosensory cortex, insula, ACC

## Abstract

First- and third-person experiences of bodily sensations, like pain and touch, recruit overlapping neural networks including sensorimotor, insular, and anterior cingulate cortices. Here we illustrate the peculiar role of these structures in coding the sensory and affective qualities of the observed bodily sensations. Subsequently we show that such neural activity is critically influenced by a range of social, emotional, cognitive factors, and importantly by inter-individual differences in the separate components of empathic traits. Finally we suggest some fundamental issues that social neuroscience has to address for providing a comprehensive knowledge of the behavioral, functional and anatomical brain correlates of empathy.

## Introduction

We refer to empathy as that fundamental process in human social interactions that allows the understanding of others people sensations and emotions by sharing their sensory and affective states. However, despite philosophers, developmental and social psychologists having long investigated empathy (Eisenberg et al., 1987; Batson, 1991; Eisenberg, 2000; Hoffman, 2000), there is still no universal agreement on its definition and on the different interrelated phenomena it subsumes (a review of this debate: Preston and de Waal, 2002; Blair, 2005a; de Vignemont and Singer, 2006; Batson, 2009). Numerous scholars suggested that empathy comprises several components and independent but interacting mechanisms (Davis, 1996; Eisenberg, 2000; Decety and Jackson, 2004), such as sensory-affective and emotional sharing (Preston and de Waal, 2002), cognitive perspective taking of others’ states (Davis, 1996; Decety and Jackson, 2004), the ability to discern the other as the source of our own affective state (review in Singer and Lamm, 2009) and self-regulatory mechanisms that influence the extent of the empathic experience and the likelihood of prosocial behaviors (see Decety, 2011 for a critical discussion). Indeed, empathic reactions may stem from feelings of sorrow for others’ pain (i.e., sympathy) to distress for an unpleasant scene (Batson, 1991; Davis, 1996).

Social neuroscience has only recently started to investigate the neural underpinnings of empathy being strongly influenced by the shared representation accounts which postulate that the human ability to understand others’ motor, perceptual, and emotional states is sub-served by the activation of corresponding representations in the observer (Preston and de Waal, 2002; Gallese, 2003). At the neural level, such ability could rely on mirror-like mechanisms similar to the mirror neurons that (in primate brain) encode both executed and observed actions (di Pellegrino et al., 1992). Accordingly, since their discovery numerous studies in humans found shared neural representations between self and others in the domain of actions (Rizzolatti et al., 2001; Avenanti et al., 2013; Tidoni et al., 2013), emotions (Wicker et al., 2003; Bastiaansen et al., 2009; Borgomaneri et al., 2012) and sensations, like pain and touch (Keysers et al., 2010; Bernhardt and Singer, 2012).

Here we focus on the brain regions involved in first- and third-person experience of pain and touch, and illustrate their peculiar role in coding the sensory and affective qualities of these bodily sensations. Subsequently we show how—despite such vicarious activations seeming to occur automatically (i.e., without conscious and effortful processing)—they can be modulated by inter-individual differences in personality traits, dispositions, attitudes, and social and cognitive forms of interpersonal evaluation of the other. We conclude by suggesting that some fundamental issues have to be addressed by future research to improve knowledge on the complex relationship between behavioral and both functional and anatomical neural correlates of empathy.

## Vicarious neural activations to others’ pain and touch

### Vicarious Pain

Experiencing pain involves two complementary but dissociable components (Craig, 2002) encoded in distinct nodes of the so-called “pain matrix” neural network (Melzack, 1999). The sensory discriminative component concerns the physical qualities of the stimulus (e.g., intensity) and is associated with activity in somatosensory and motor cortices. The affective-motivational component relates to the subjective aspects of pain perception (e.g., unpleasantness) and is encoded by the anterior insula (AI), which is known to be involved in representing and integrating internal and emotional feelings states (Craig, 2002) and by the anterior cingulate cortex (ACC) (Peyron et al., 2000), which is known to re-represent the emotional global states to control, select, and prepare appropriate responses (Medford and Critchley, 2010).

Yet, pain perception is not only a private state. Understanding others’ pain is a fundamental ability in social interactions that is sub-served by the same neural structures as those involved in first-person experience of pain (Preston and de Waal, 2002; Gallese et al., 2004; Keysers and Gazzola, 2009; Decety, 2011). The sensory discriminative aspects of observed pain are associated with activity in primary (S1) and secondary (S2) somatosensory cortices (Bufalari et al., 2007; Saarela et al., 2007; Costantini et al., 2008; Valeriani et al., 2008; Akitsuki and Decety, 2009; Betti et al., 2009; Voisin et al., 2011; Aziz-Zadeh et al., 2012), as well as in primary motor cortex (M1) (Avenanti et al., 2005), while the affective-motivational qualities of observed pain are associated with activity in AI and ACC (Morrison et al., 2004; Singer et al., 2004; Botvinick et al., 2005; Jackson et al., 2005; Singer et al., 2006; Saarela et al., 2007). Empathic responses in these regions could thus reflect a process that represents bodily and affective states in the self and in the others, with the final aim to guide homeostatic and behavioral responses (Singer and Lamm, 2009).

### Vicarious Touch

Observing touch also elicits mirror-like responses. Increasing evidence points to the peculiar role of somatosensory cortices in processing sensory qualities of observed touch (Keysers et al., 2010; Morrison et al., 2011a). S2 is active both when being touched and observing someone else being touched (Keysers et al., 2004; Blakemore et al., 2005; Schaefer et al., 2006; Ebisch et al., 2008). Functional neuroimaging (Blakemore et al., 2005; Schaefer et al., 2009, 2012) and electroencephalography (Bufalari et al., 2007; Martinez-Jauand et al., 2012) studies showed that also S1 responds to observed touch, especially when the body is seen from a first-person perspective (Schaefer et al., 2009) and its activity (as indexed by early Somatosensory Evoked Potentials (SEPs)) correlates with intensity (but not with the unpleasantness) of the observed bodily sensations (Bufalari et al., 2007).

## The social neuroscience of empathy for pain and touch

### Social Pain

Fascinatingly, rather than being fixed, the empathic behavioral and neural responses can be reduced or increased by a broad range of cognitive (Lamm et al., 2007a), emotional, and social factors (de Vignemont and Singer, 2006), such as personal state and interpersonal relationship or appraisal of the other in pain.

For example, in acupuncturists—who must prevent distress to impair their ability to be of assistance—the ACC and AI neural responses to others’ pain are significantly reduced (Cheng et al., 2007). Similarly, being in pain oneself while observing pain in others may reduce the vicarious activity of the somatic nodes of the pain matrix (Valeriani et al., 2008), suggesting that being in pain may bias the empathic relation with others towards self-centered empathic stances.

On the other hand, adopting the perspective of a beloved person in pain increases activity in ACC and AI (Cheng et al., 2010). Conversely, affective sharing of pain of an unfair other is associated with reduced fronto-insular and ACC activity and increased activation of reward-related areas (Singer et al., 2006). Social in/out group membership can also modulate the brain activity related to agonistic or antagonistic motivation to empathize and to pro/antisocial behavior. Indeed, other-oriented feelings of sympathy and AI activity predicted the tendency to engage in costly behavior to reduce an affiliated soccer fan’s pain, while subjective negative evaluations of the opponent fan and nucleus accumbens activations predicted the tendency to not make a sacrifice for this individual (Hein et al., 2010). Similarly, observing members of different ethnicity being in pain reduces the sensorimotor empathic response (Avenanti et al., 2010), while observing pain experienced by own- versus other-race individuals increases autonomic reactivity, ACC and AI activity (Xu et al., 2009; Azevedo et al., 2012) as a function of the observers’ implicit racial biases (Avenanti et al., 2010; Azevedo et al., 2012; Sessa et al., 2013). The behavioral and neural empathic resonance can also be modulated by a priori attitudes toward the target group. Indeed, empathy ratings, AI and midcingulate activity are stronger for the observation of pain in HIV/AIDS transfusion targets, but weaker for HIV/AIDS drug targets (Decety et al., 2010).

Thus, empathic resonant activity in empathy-related neural networks may interact with (and be modulated by) the activity of other neural networks relevant for social cognition such as those involved in mentalizing, in coding reward, or in cognitive control and emotion regulation.

### Social Touch

The affective and social meaning of touch can modulate behavioral and neural responses to observed human tactile interactions. Indeed skin-to-skin contact is crucial for social interactions sub-serving nonverbal communication of intentions and emotions. Observing a face being touched by fingers enhanced the detection of around-threshold tactile stimuli on the observer’s face (Cardini et al., 2011), more strongly if the observers and the observed faces belong to the same (versus different) social group (Serino et al., 2009).

Also, the affective meaning conveyed by a hand stroking a body increases S1, S2, and insular activity (Morrison et al., 2011a). Particularly, S1 activity is stronger when observing human-based intentional touch (Ebisch et al., 2008) and is causal to understanding the affective consequences (Bolognini et al., 2013) of tactile interactions between people (Rossetti et al., 2012). Even when touch is physically experienced, S1, S2, and insular activity are stronger when participants receive a gentle stroking performed by a hand (with respect to a stick; Kress et al., 2011). Interestingly, S1 activity is further modulated by the believed (opposite) gender of the caresser, despite the sensory stimulation properties being the same across genders (Gazzola et al., 2012). These results highlight the twofold function of S1 in social interactions: it encodes the sensory qualities of first- and third-person experience of bodily sensations, and is further modulated by the attributed affective components of human tactile interactions. Modulation of S1 activity related to somatic and affective qualities of observed sensations is probably due to feedback projections from multimodal fronto-parietal (Macaluso and Driver, 2005) and insula areas. Indeed, processing gentle touch and its associated pleasant sensation is conveyed by the so-called tactile C (CT) fibers (Olausson et al., 2002), which project to the insular cortex (Bjornsdotter et al., 2010) that in turn is functionally connected to the sensorimotor cortices (Deen et al., 2011). Pathologically reduced CT-fiber density is associated with a less pleasant evaluation of observed interpersonal touch, and with absent modulation of insular activity (Morrison et al., 2011b). Conversely, in healthy participants the observation of somebody else’s arms being stroked elicits a similar response in the posterior insula as when one is directly feeling touch (Morrison et al., 2011a). These results suggest that the representations of our feeling states in insula form the basis for understanding the feelings of others. Ebisch et al. (2011) found opposite activation patterns in posterior insula for first- and third-person experience of affective human touch and suggested this region can differentiate the stimulation source (self versus other), which is consistent with its role in mediating the sense of body ownership (Heydrich and Blanke, 2013).

## The personality neuroscience of empathy for pain and touch

### Empathic Traits

Empathic responses comprise cognitive, affective, and emotional components (Batson, 1991), and may reflect stable personality dispositions (trait empathy; Davis, 1996) or be linked to situational and contextual factors (state empathy; Batson et al., 1983). From a neuroscientific perspective this suggests that distinct neural mechanisms may underpin different types of empathy-related responses.

Indeed, empathy-related activity in the affective division of the pain matrix correlates with scores in trait empathy emotional scales (Singer et al., 2004; Lawrence et al., 2006; Lamm et al., 2007a; Saarela et al., 2007; Cheetham et al., 2009; Lang et al., 2011), such as the Balanced Emotional Empathy Scale (Mehrabian and Epstein, 1972), the Emotional Contagion Scale (ECS; Doherty, 1997), and both the Empathic Concern (EC) and Personal Distress (PD) subscales of the Interpersonal Reactivity Index (IRI; Davis, 1996). However, a recent meta-analysis suggested that empathic neural responses are better predicted by situational rather than by dispositional measures of emotional empathy (Lamm et al., 2011).

Also the empathy-related activity in the sensory division of the pain matrix shows a complex pattern of correlations with different empathic components. The empathic sensorimotor response is independently predicted not only by the sensory qualities of pain, but also: (i) positively by the participants’ ability to imaginatively transpose into others’ feelings and states (as indexed by IRI-PT subscale); and (ii) negatively by either the situational than the stable tendencies to experience personal distress as a result of others’ pain (Avenanti et al., 2009). Interestingly, also vicarious pain-related activity in S1 is positively correlated with IRI-PT scores (Cheng et al., 2008; Martinez-Jauand et al., 2012). Additionally, functional and anatomical neuroimaging studies showed significant correlations between self-oriented emotional empathy (as indexed by IRI-PD) and (i) vicarious sensorimotor activations to others’ pain (but only in females: Yang et al., 2009), and (ii) reduced gray matter volume in S1 (Banissy et al., 2012). These results thus suggest that both brain structure and vicarious activity in the sensory node of the pain matrix are independently influenced by distinct functional, not purely sensory, mechanisms.

Remarkably, the role exerted by inter-individual differences in cognitive empathy has been demonstrated also for touch-related vicarious activity in S1. PT scores are positively correlated with increased amplitude of early SEPs (Martinez-Jauand et al., 2012), S1 hemodynamic responses to observed touch (Schaefer et al., 2012), and impairments in encoding the affective valence of others’ somatic feelings resulting from disruption of S1 activity (Bolognini et al., 2013). No associations, instead, have been reported between vicarious somatosensory activations to touch/pain and other trait cognitive (IRI-Fantasy Scale) or emotional empathy scales [IRI-PD, IRI-EC, Empathic Quotient (Baron-Cohen and Wheelwright, 2004) or ECS (Doherty, 1997)]. Interestingly, similarly to the domain of sensations PT—but not other IRI subscales—correlates also with S1 vicarious activity to heard human actions (Gazzola et al., 2006).

Thus, taking into account that different experimental designs and manipulations were used, it seems that a rather coherent picture emerges from the above-mentioned studies. Indeed, structures coding affective qualities of observed sensations are more closely related to emotional empathy traits, while vicarious activity in structures coding sensory qualities of observed sensations is differentially modulated by cognitive perspective taking abilities and self-oriented empathic responses.

### The influence of the social, affective and emotional abilities on empathy

The behavioral and neural empathic responses have been recently investigated in pathological conditions affecting the social and emotional sphere, as well as in participants with different affective styles.

Clinical studies indicate that psychopaths show cognitive empathy and mentalizing abilities in the normal range (if not higher) but they lack emotional reactivity and sympathy responses (Blair, 2005b). Autistics, instead, show reduced theory of mind and metalizing-related brain activity (Frith and Frith, 2006). Interestingly, the sensorimotor response to others’ pain is greater in (healthy) subjects with high scores in a psychopathology scale (Fecteau et al., 2008) and absent in individuals with Asperger syndrome (Minio-Paluello et al., 2009).

Based on the assumption that awareness of one’s own emotional states is a prerequisite for recognizing such states in others (Decety and Jackson, 2004), alexithymic patients—who have a deficit in identifying and expressing one’s own emotional states—show reduced ACC activations to others’ pain, and score low in empathy questionnaires (Moriguchi et al., 2007). Also, alexithymic scores of control participants are negatively correlated with left AI activity during imagination of a close other in pain (Bird et al., 2010). Interestingly, insular response to the observation of a beloved in pain is also associated with the tendency to regulate one’s own emotional responses on the base of bodily-emotional states (Mazzola et al., 2010), i.e., with “inward” dispositional affective style (Arciero et al., 2004).

These results thus confirm that representations of our bodily and emotional feeling states in insula and ACC form the basis for understanding and reacting to the feelings of others.

## Future perspectives

Recent theoretical and methodological advances in social and cognitive neuroscience critically improved the conceptualization of neurocognitive models of human empathy. Future studies might fruitfully address some fundamental issues on the relationship between behavioral, functional and anatomical brain correlates of empathy.

One important issue regards the causal nature of the relationship between empathy-related behavior and brain activity. Further studies are needed to show whether changes in empathy-related brain activity—as induced by brain stimulation techniques (such as TMS or transcranial Direct Current Stimulation)—can change empathic behavioral responses, as well as changes in empathic behavior—as induced by focused training or psychotherapy—can induce changes in empathy-related brain activity. Initial findings suggest a bidirectional influence by showing that (i) interfering/enhancing the activity of empathy-related brain structures produces impairments/enhancements in empathy tasks and traits (Balconi and Bortolotti, 2012; Rossetti et al., 2012; Bolognini et al., 2013), while (ii) focused training on empathic resonance increased vicarious activity in affective node of the pain matrix when witnessing people suffering (Klimecki et al., 2013).

An additional major issue is the association between anatomical and functional brain organization related to empathic personality features. Recent evidence shows that the same regions (in particular ACC and AI) were identified by both functional and structural neuroimaging as the neural substrate of specific empathic traits (Yang et al., 2009; Banissy et al., 2012). Despite the indication that structural and functional changes can be associated (Durston and Casey, 2006), the work on the relationship between anatomical and functional features of empathy is still very limited, and the conclusions have to be considered with caution.

A third main issue regards the relationship between personality dimensions, empathic traits, and vicarious brain activations to others’ emotions and sensations. Despite it being known that different personality factors individuated by the Big Five theory of personality (McCrae and Costa, 1991) are related to distinct empathic components (EC is closely related to agreeableness, PD to neuroticism, while PT shows a complex interstitial relationships with the 5 factors; Mooradian et al., 2011), there are still limited data concerning this relationship (Marcoux et al., 2013; Schaefer et al., 2013). Gender also plays a role in this complex relationship. Indeed, women have higher empathic abilities, neuroticism, agreeableness, and extraversion scores (Goodwin and Gotlib, 2004), and seem to have also stronger vicarious-pain-related brain activations (Han et al., 2008; Yang et al., 2009). However, studies investigating the interplay between personality, gender, and empathy-related brain activity are still lacking and should involve highly representative samples, larger than those commonly used in neuroimaging experiments.

In summary the available data have enhanced the understanding of vicarious experience at both neural and psychological levels. However, in order to fulfill the needs of a comprehensive and predictive model of human empathy, further work will have to integrate converging evidence from the molecular, cellular, and systemic levels both in healthy and neurological conditions.

## Conflict of interest statement

The authors declare that the research was conducted in the absence of any commercial or financial relationships that could be construed as a potential conflict of interest.
